# Articular involvement, steroid treatment and fibromyalgia are the main determinants of patient-physician discordance in systemic lupus erythematosus

**DOI:** 10.1186/s13075-020-02334-5

**Published:** 2020-10-14

**Authors:** Elena Elefante, Chiara Tani, Chiara Stagnaro, Viola Signorini, Alice Parma, Linda Carli, Dina Zucchi, Francesco Ferro, Marta Mosca

**Affiliations:** 1 Rheumatology Unit, Department of Clinical and Experimental Medicine, University of Pisa, Via Roma 67, Pisa, Italy; 2 Department of Medical Biotechnologies, University of Siena, Siena, Italy

**Keywords:** Systemic lupus erythematosus, Patient-reported outcomes, Patient-physician discordance, LLDAS, Disease burden, HRQoL

## Abstract

**Background:**

Remission or the lowest possible disease activity is the main target in the management of systemic lupus erythematosus (SLE). Anyway, conflicting data are present in the literature regarding the correlation between physician-driven definitions and patient perception of the disease. The objective of this study is to evaluate the relationship between the definition of lupus low disease activity state (LLDAS) and patient’s health-related quality of life (HRQoL).

**Methods:**

This is a cross-sectional, monocentric study. Adult SLE patients were included. For each patient, demographics, disease duration, medications, comorbidities, organ damage, active disease manifestations and SELENA-SLEDAI were assessed. Patients have been categorised as follows: LLDAS, remission and active disease. Each patient completed the following patient-reported outcomes (PROs): SF-36, LIT, FACIT-Fatigue and SLAQ. A SLAQ score < 6 (25° percentile of our cohort) was used as the cut-off value to define a low disease activity state according to patient self-evaluation.

**Results:**

We enrolled 259 consecutive SLE patients (mainly female and Caucasian, mean age 45.33 ± 13.14 years, median disease duration 14 years). 80.3% were in LLDAS, of whom 82.2% were in remission; 19.7% were active. No differences emerged for any of the PROs used between the LLDAS and the active group.

Considering the LLDAS subgroup, we identified 56 patients with a subjective low disease activity (SLAQ < 6) and we defined them as “concordant”; the remaining 152 patients in LLDAS presented a subjective active disease (SLAQ ≥ 6) and were defined “discordant”. Discordant patients presented more frequently ongoing and past joint involvement (*p* < 0.05) and a diagnosis of fibromyalgia (*p* < 0.01); furthermore, they were more likely to be on glucocorticoid therapy (*p* < 0.01). Discordant patients showed a significantly poorer HRQoL, assessed by all PROs (*p* < 0.0001).

**Conclusions:**

Joint involvement, glucocorticoid therapy and comorbid fibromyalgia resulted to be the most important variables determining the poor concordance between patient and physician perspective on the disease.

## Background

The main targets of systemic lupus erythematosus (SLE) management are considered to be as follows: attainment of disease remission or the lowest possible level of disease activity, prevention of disease flares and prevention of organ damage [1]. Indeed, remission and a low disease activity state (LLDAS) are linked to better outcomes in terms of organ damage preservation [2], fewer incidences of hospitalisation, improved mortality and better quality of life [3, 4]. Recent studies have demonstrated an association between remission or LLDAS achievement and better health-related quality of life (HRQoL) in SLE patients, although this may sometimes prove to be a weak association [5, 6]. Crucially, HRQoL improvement is considered to be a major outcome measure in clinical trials for the development of new drugs and represents an ideal treatment target in SLE clinical practice.

However, literature data also show that traditional clinical indicators for disease activity and/or organ damage possess only a weak correlation to quality of life (QoL) measures, suggesting that such measures assess different aspects of patient status. This may be due to the existence of discrepancies between patient and physician assessment of SLE disease status. For instance, the literature shows some evidence that patients may not be aware of clinically important signs of disease activity if they are not accompanied by symptoms, such as haematological abnormalities or proteinuria, in spite of the fact that the prime focus of physicians is on major organ manifestations.

Moreover, a patient’s QoL is influenced not only by clinical disease manifestations but also by psychosocial aspects such as demographic and socioeconomic factors, comorbidities, fibromyalgia, fatigue and mood disorders. Such factors are considered the main determinants of QoL in SLE [7–9]. Definitions of remission and LLDAS are physician-driven definitions and conflicting data are present in the literature regarding their correlation with the patient’s disease perception.

The aim of this study is therefore to evaluate the relationship between the current definition of LLDAS and the patient’s perspective on disease burden and quality of life.

## Methods

This is a cross-sectional, monocentric study conducted at the Rheumatology Unit of the University of Pisa. Participants were consecutive adult in- and out-patients with a SLE diagnosis (according to the 1997 ACR classification criteria), and all regularly attended our Lupus Clinic. Patients were enrolled in this study between December 2018 and July 2019.

The study was approved by the local ethics committee, with patients signing informed consent.

We retrieved data from clinical records regarding epidemiologic and demographic characteristics, disease duration, cumulative organ involvement, comorbidities and concomitant medications. At the time of enrollment, active disease manifestations and laboratory tests were evaluated in order to assess disease activity using the Safety of Estrogens in Lupus Erythematosus National Assessment-Systemic Lupus Disease Activity Index (SELENA-SLEDAI) [10]. Organ damage was assessed using the SLICC-Damage Index (SLICC-DI) [11]. The remission status of patients at enrollment was defined according to the European consensus criteria (DORIS) [12]. LLDAS was defined according to the Asian Pacific Lupus Consortium definition [13]. For patients with fibromyalgia, the diagnosis was based on the ACR 1990 classification criteria [14].

At enrollment, each patient completed the following patient-reported outcomes (PROs):

- The SF-36 (Medical Outcomes Study Short Form 36) questionnaire to assess patients’ HRQoL. This questionnaire addresses eight areas and can be summarised into two global scores: the physical component summary (PCS) and the mental component summary (MCS). Each score ranges from 0 to 100, with higher values representing better self-perceived HRQoL [15, 16].
- The Lupus Impact Tracker (LIT) questionnaire to assess the impact of SLE on daily living which includes 10 questions about cognition, lupus medication, physical health, pain/fatigue impact, emotional health, body image and planning/desires/goals. This questionnaire provides a single summary score from 0 to 100, with lower scores signifying lower impact of SLE on patient life [17].
- The FACIT-Fatigue questionnaire to assess fatigue. FACIT-Fatigue assesses fatigue in the physical, emotional, functional, social and daily living domains. The score ranges from 0 to 52, with lower scores indicating greater fatigue [18, 19].
- The Systemic Lupus Activity Questionnaire (SLAQ), which is a patient-reported assessment of subjective SLE disease activity. It was developed on the basis of the SLAM [20]. The SLAQ score includes a list of questions regarding 24 symptoms including constitutional, mucocutaneous, articular, respiratory, neuropsychiatric and gastrointestinal manifestations. Patients are asked to choose between four options for each symptom: (a) mild, (b) moderate, (c) severe and (d) no symptom. Items are weighted according to their clinical importance and grouped into 17 categories; each item can be scored from 0 to 3 according to the severity of the symptom. The final SLAQ score sum can range from 0 to 47. The Italian version of the SLAQ questionnaire has been validated by Tani et al. [21]. Patients included in this analysis were categorised in the following groups: remission, on or off treatment, LLDAS and active disease status.

Given the lack of a recognised SLAQ score value which could function as a cut-off point for self-perceived disease activity, we empirically agreed on a SLAQ score < 6, corresponding to the 25° percentile of our cohort, as the cut-off value to define a low disease activity state according to patient self-evaluation when considering the distribution of the SLAQ scores in our cohort.

### Statistical analysis

Continuous data have been reported as median and interquartile range (IQR) or as mean and standard deviation (SD) as appropriate. Categorical data have been reported as a percentage. The Student *t* test, Mann-Whitney and chi-square tests have been conducted for univariate analysis.

The Spearman test has been used for linear correlation between continuous data. Multivariate analysis has also been performed by multiple linear and logistic regression for variables which were significantly associated within univariate analysis.

All *p* values less than 0.05 have been considered statistically significant. Statistical analysis has been performed using STATA-13 software.

## Results

We enrolled 259 consecutive SLE patients who were predominantly female (93.05%) and of Caucasian ethnicity (97.2%). The median age of the cohort at enrollment was 46 years (IQR 36–54) and median disease duration was 14 years (IQR 6–21).

### The physician’s perspective

Table 1 reports the clinical data, ongoing treatment and comorbidities of the study population.

**Table 1:** Epidemiologic and clinical baseline characteristics of the cohort

	LLDAS (208/259, 80.3%)	Active (51/259, 19.7%)	***p*** value
Median age at enrollment (years) (IQR)	47 (38.5–55)	38 (30–47)	***p*** **< 0.001**
Median disease duration (years) (IQR)	15 (7–21)	9 (5–17)	*p* = 0.052
Ethnicity (% of Caucasian)	98.6%	96.1%	ns
Sex (% of female)	92.3%	96.1%	ns
Median SELENA-SLEDAI at enrollment (IQR)	2 (0–2)	6 (5–8)	***p*** **< 0.0001**
Median SLICC-DI at enrollment (IQR)	1 (0–2)	0 (0–1)	ns
Fibromyalgia (% of patients)	12%	5.9%	ns
Cumulative renal involvement (% of patients)	42.8%	47%	ns
Cumulative cutaneous involvement (% of patients)	51.9%	66.7%	*p* = 0.062
Cumulative articular involvement (% of patients)	66.3%	76.5%	ns
Cumulative serositic involvement (% of patients)	19.2%	19.6%	ns
Cumulative haematological involvement (% of patients)	49%	66.7%	***p =*** **0.026**
Cumulative neuropsychiatric involvement (% of patients)	12.5%	7.8%	ns
Ongoing GC (% of patients)	44.2%	78.4%	***p*** **< 0.001**
Median daily GC dose (mg methylprednisolone) (IQR)	0 (0–4)	4 (2–8)	***p*** **< 0.0001**
Ongoing immunosuppressant (% of patients)	39.4%	62.7%	***p =*** **0.003**
Ongoing HCQ (% of patients)	78.4%	76.5%	ns

The *lupus low disease activity state* (LLDAS) definition and the DORIS definition for remission have been applied in our consideration of clinical and serological disease activity and ongoing treatment. Two hundred eight of 259 (80.3%) patients satisfied the LLDAS definition, of whom 171 patients were in remission (on or off treatment); the remaining 51/259 patients (19.7%) failed to meet the LLDAS definition or remission and were therefore considered active.

The most frequent types of disease manifestation in the active patients were as follows: renal (19/51, 37.2%), cutaneous (16/51, 31.4%), articular (15/51, 29.4%) and haematological (14/51, 27.4%). None of the patients presented with active neuropsychiatric lupus (NPSLE) at enrollment. The most frequent disease manifestations in the LLDAS patients were as follows: haematological (23/208, 11%), mainly mild leukopenia; articular (16/208, 7.7%); and cutaneous (12/208, 5.8%).

When comparing the two groups of patients identified according to the definitions of LLDAS (including remission) and active disease, we found that active patients were significantly younger than LLDAS patients (*p* < 0.001) and had a slightly shorter disease duration (*p* = 0.052). No differences emerged for the other epidemiologic characteristics (sex, ethnicity). As expected, the median SLEDAI score differed significantly between groups (*p* < 0.0001); a higher percentage of active patients was treated with glucocorticoids (GC) (*p* < 0.001) and immunosuppressive treatment (*p* < 0.01) compared to LLDAS patients, while no differences emerged regarding the SLICC-DI. In terms of cumulative organ involvement, the only difference was that past haematological manifestations were significantly more frequent among active patients than LLDAS patients (*p* < 0.05). Finally, there were no evident differences in terms of the percentage of patients with concomitant fibromyalgia. Table 1 summarises these findings.

### The patient’s perspective

Table 2 shows the results of the univariate analysis of patient-reported outcomes (PROs) in the two subgroups of patients with active disease and LLDAS. We found no statistically significant differences for any of the questionnaires used (SF-36, LIT, FACIT-Fatigue, SLAQ) when we compared the PRO results of the LLDAS group and the active group of patients.

**Table 2:** Results of the univariate analysis of PROs in LLDAS vs active patients

	LLDAS (208/259, 80.3%)	Active (51/259, 19.7%)	***p*** value
PCS (mean ± SD)	57.8 ± 16.8	59.8 ± 15.8	ns
MCS (mean ± SD)	56.9 ± 16.1	57.6 ± 15.9	ns
LIT (mean ± SD)	24.5 ± 20.5	28.5 ± 19.9	ns
FACIT (mean ± SD)	38.6 ± 10.3	36.7 ± 10.6	ns
SLAQ (mean ± SD)	10.8 ± 7.4	12.4 ± 7.6	ns

In the multivariate analysis, after adjusting for confounding factors (age at enrollment, GC and immunosuppressive therapy), only the LIT (*p* = 0.042) and the SLAQ (*p* = 0.032) scores showed significantly lower scores in the LLDAS group compared to the active group.

### Patient-physician discordance

We identified 56 patients in the subgroup of 208 LLDAS patients with a subjective condition of low disease activity (SLAQ < 6) and defined such patients as “concordant”; conversely, the remaining 152 LLDAS patients presented a subjective active disease (SLAQ ≥ 6) and we defined such patients as “discordant”.

We subsequently compared these two subgroups of LLDAS patients (concordant vs discordant) in order to identify contributing factors to patient-physician discordance (Table 3).

**Table 3:** Determinants of patient-physician discordance

	Discordant patients (152/208)	Concordant patients (56/208)	***p*** value
Median age at enrollment (years) (IQR)	48 (39–56)	44.5 (37–50)	*p =* 0.031
Median disease duration (years) (IQR)	16.5 (7.75–22.25)	13.5 (5–19)	ns
Median SLICC-DI (IQR)	0 (0–1)	1 (0–2)	ns
Ongoing joint involvement (% of patients)	10.5%	0	***p = 0.012***
Past joint involvement (% of patients)	70.9%	55.3%	***p = 0.036***
GC therapy (% of patients)	50%	28.6%	***p = 0.006***
Median GC daily dose (mg methylprednisolone) (IQR)	0 (0–2)	1 (0–4)	***p =*** **0.031**
Fibromyalgia (% of patients)	15.8%	1.8%	***p = 0.006***
PCS (mean ± SD)	54.7 ± 14.1	65.8 ± 20.4	***p*** **< 0.0001**
MCS (mean ± SD)	54 ± 14.5	64.3 ± 17.7	***p*** **< 0.0001**
LIT (mean ± SD)	28.4 ± 20.4	14 ± 16.8	***p*** **< 0.0001**
FACIT (mean ± SD)	35.3 ± 10.1	47.3 ± 3.6	***p*** **< 0.0001**

We found that concordant patients were younger compared to discordant patients (*p* < 0.05), while no significant differences emerged between the two subgroups in terms of disease duration and organ damage (SLICC-DI). Discordant patients presented more frequently with ongoing and past joint involvement (*p* < 0.05); there were no differences concerning other organ manifestations.

Furthermore, discordant patients were more likely to have a diagnosis of concomitant fibromyalgia (*p* < 0.01) than concordant patients, as well as being more likely to be on glucocorticoid therapy (*p* < 0.01); however, there were no differences between the two subgroups in terms of immunosuppressants and hydroxychloroquine (HCQ) treatment.

As expected, discordant patients showed a significantly poorer quality of life, assessed by all PROs (*p* < 0.0001). In particular, these patients reported lower scores of PCS, MCS and FACIT-Fatigue and higher scores of the LIT questionnaire, suggesting that patients who judge their disease as active, despite being in LLDAS, also have a poorer quality of life (Table 3).

## Discussion

Prognosis of SLE patients has greatly improved in recent decades, thanks to early diagnosis, advances in treatment strategies and better management of comorbidities. However, such advances have not always been coupled with similar improvements in patients’ quality of life (QoL) [22]. It is well known that HRQoL of SLE patients is still consistently lower than that of matched healthy subjects [22], as well as being lower than that of patients with other chronic rheumatic diseases [23, 24].

The 2019 EULAR recommendations for SLE management defined optimisation of HRQoL as one of its treatment goals [25]. Remission or low disease activity represents the ideal treatment goal for SLE patients; however, conflicting data are present in the literature regarding the impact of such disease targets on the improvement of HRQoL.

The aim of this study of a large monocentric cohort of SLE patients was to investigate whether or not the attainment of a condition of low disease activity correlates with a subjective perception of well-controlled disease, a lower perceived disease burden and a better HRQoL from the patient’s perspective.

The majority of patients enrolled in our cohort fulfilled the definition of LLDAS (80.3%); furthermore, the majority also satisfied the DORIS definition of remission in SLE, therefore suggesting that these definitions represent achievable targets for SLE patients in routine clinical practice.

LLDAS patients and patients with active disease in our cohort presented similar epidemiologic and demographic characteristics, except for a younger age at enrollment of active patients (*p* < 0.001). Moreover, we found no significant differences between the two groups in terms of HRQoL and perception of fatigue. This suggests that being in LLDAS was not associated with a better QoL in our cohort. However, it is interesting to note that the SLE-specific questionnaire LIT, which constitutes a more thorough investigation of different aspects of disease impact on patient life, was able to identify a difference in disease burden perception between active patients and LLDAS patients, with lower scores in the LLDAS group.

Although the relationship between disease activity and HRQoL in SLE is still controversial [9, 26, 27], some recent data from the literature would seem to demonstrate a positive impact of the conditions of remission/LLDAS on patients’ HRQoL [28], in contrast with our findings. In any case, the results of some of these recent studies are not comparable with this study due to some important differences in the populations included in contrast with our cohort.

For example, Golder et al. demonstrated in a large cohort of SLE patients that the attainment of a condition of LLDAS correlates with a better QoL, evaluated by the SF-36, even after adjustment for other variables associated with HRQoL. In any case, the study’s cohort included patients primarily of Asian ethnicity, with the small percentage (8%) of Caucasian patients enrolled showing a poorer QoL [29]. Ugarte et al. demonstrated an association between the state of remission or low disease activity and better SF-36 scores in a multiethnic US cohort (LUMINA cohort). Only 28% of patients were Caucasians, with the majority being African and Hispanic. Moreover, enrolled patients had a significantly shorter disease duration compared to our study (1.4 vs 14 years). Finally, the authors used different definitions of remission and low disease activity for their study according to which a stable immunosuppressive treatment was not included in the conditions of remission or low disease activity [30]. The impact of remission duration on patient’s quality of life is well exemplified by Mok et al. whose study, conducted on a large cohort of SLE Chinese patients with a mean disease duration of 12.6 ± 8.1 years, demonstrated that a durable remission could be achieved in almost a quarter of patients; however, only patients with remission of ≥ 5 years presented a significantly better QoL assessed using both SF-36 and Lupus-PRO [31]. In terms of the LIT questionnaire, which has nevertheless demonstrated a sound correlation with disease activity evaluation (SELENA-SLEDAI and physician global assessment—PGA) in the validation studies conducted in Europe and the USA [32, 33], there is a lack of data concerning correlation with the definitions of LLDAS or remission in SLE. Considering that patients in our longstanding cohort with a well-controlled disease failed to report a better HRQoL compared to patients with active disease, we tried to identify which particular clinical factors might have the greatest influence on perception of health status among LLDAS patients, in turn leading to a different view of the disease on the part of the patient compared to their physician.

Considering the SLAQ scoring system and the distribution of the SLAQ results in our cohort, we empirically identified two subgroups of LLDAS patients from the SLAQ questionnaire results (patients’ self-evaluation of disease activity): namely, concordant patients (with a subjective low disease activity state defined as SLAQ < 6) and discordant patients (those with a subjective active disease, defined as SLAQ ≥ 6). The majority of LLDAS patients (151/208) was in the discordant subgroup, suggesting that most patients with a good control of disease activity according to the clinician actually viewed their disease as active.

Furthermore, we found that the main determinants of patient-physician discordance in SLE patients in LLDAS were past and ongoing joint involvement, a concomitant diagnosis of fibromyalgia and ongoing GC treatment, due to the fact that they were reported in the discordant subgroup with a significantly greater frequency.

Such findings are in line with data from the literature which demonstrates that joint involvement and chronic pain constitute some of the most important determinants of QoL in SLE [34]. Interestingly, Piga et al. have demonstrated that musculoskeletal manifestations, including active arthritis as well as Jaccoud’s deformities and fibromyalgia, are associated with a poorer HRQoL (as measured by SF-36) and a negative disability perception (evaluated by the HAQ) in SLE patients [35]. Similarly, we have previously demonstrated that active arthritis proved to be significantly correlated with VAS score for pain and patient perception of disease activity and global health [36]. Fibromyalgia is another well-known factor independently associated to a poorer HRQoL in SLE patients [7] and strongly associated with fatigue, one of the most common and debilitating symptoms for SLE patients [37, 38]. SLE is a complex chronic disease; evidence from the literature and experience from clinical practice suggest that a discordance exists between patient and physician perspectives. A recent study by Golder et al. revealed that the three highest-ranked patient concerns (all of which related to milder manifestations with substantial impact on functionality) were not routinely assessed by the majority of physicians, highlighting a significant gap in healthcare communication [39]. Alarcòn et al. compared patient and physician assessment of disease activity (through a 10-cm anchored visual analogue scale) in a multiethnic cohort of 300 SLE patients. Their study found that patients and physicians rate disease activity in SLE differently; indeed, there was a discrepancy in 58% of patients. In particular, poor self-perceived functioning and joint involvement were positively associated to the discrepancy in this study [40]. Similarly, Yen et al. found that bodily pain was the most important variable for predicting “clinically relevant” discordance in the evaluation of disease activity in a large group of SLE female patients [41]. A further important aspect requiring consideration is that the psychological status of patients may influence their self-evaluation of disease activity. Neville et al. found that the SF-36 mental health score predicted a discrepancy between patient and physician assessment of lupus activity in a cohort of Caucasian patients with longstanding SLE [42]. Crucially, our study also demonstrates that patients with low disease activity and on GC treatment were more frequently discordant from the physician’s assessment. This finding would seem to suggest that GC therapy—even at the low dosage allowed by the definition of LLDAS—contributes to patients’ perception of a poorer control of disease activity and therefore a greater disease burden.

Our final observation is that discordant patients in our cohort reported a poorer HRQoL, as measured by all the PROs used, compared to concordant patients. This underlines the fact that patient-physician discordance is strongly linked to a patient’s negative perception of their health status. Disagreement may lead to patient dissatisfaction and consequently to potentially dangerous behaviours, such as non-adherence to treatment, which ultimately determines a negative impact on disease outcomes.

We believe that the results of this study may have some implications in routine clinical practice. In particular, our findings suggest that the attainment of the target of a low disease activity does not always correspond to the patient’s perception of a well-controlled disease, particularly when arthritis persists and when the patient is on glucocorticoid therapy. Therefore, in the attempt to bridge the discordance between patients and physicians, our findings would suggest that the physician, regardless of the definition of LLDAS, should aim for optimal control of joint manifestations and to withdraw steroids, even when at low doses, whenever possible.

Our study does possess certain limitations. In particular, the cross-sectional design does not allow for the evaluation of the impact of a prolonged period of remission/LLDAS on HRQoL. Moreover, we have not performed analysis of the other characteristics of enrolled patients which might influence patient QoL. Specifically, we do not have data on the level of education, work activity status or the social condition of our patients. As those enrolled in the study were mainly out-patients, we lack patients with a severe disease even in the active group which may have flattened the differences between activity status categories.

Despite such limitations, we believe that this study has a number of strengths. The most important strength is its large patient cohort. Another strength is the fact that QoL was assessed by a variety of different questionnaires, both generic and disease-specific, to conduct a more thorough analysis of all aspects of patient daily life and disease burden.

## Conclusions

In conclusion, although LLDAS is a satisfactory treatment target for the physician, our data would seem to indicate that LLDAS may not represent the ideal goal from the patient’s perspective, particularly when low disease activity state “allows” the presence of ongoing arthritis and steroid therapy.

Therefore, better management of joint involvement and the importance of withdrawing glucocorticoids are important aspects which should be considered in SLE patient management in order to improve patient perception of health status.

## Data Availability

The datasets used and/or analysed during the current study are available from the corresponding author on reasonable request.

## Abbreviations

SLE: Systemic lupus erythematosus
LLDAS: Lupus low disease activity state
HRQoL: Health-related quality of life
QoL: Quality of life
SELENA-SLEDAI: Safety of Estrogens in Lupus Erythematosus National Assessment-Systemic Lupus Disease Activity Index
SLICC-DI: SLICC-Damage Index
PROs: Patient-reported outcomes
SF-36: Medical Outcomes Study Short Form-36
PCS: Physical component summary
MCS: Mental component summary
FACIT-Fatigue: Functional Assessment Chronic Illness Therapy
LIT: Lupus Impact Tracker
SLAQ: Systemic Lupus Activity Questionnaire
NPSLE: Neuropsychiatric systemic lupus erythematosus
HCQ: Hydroxychloroquine
PGA: Physician Global Assessment
HAQ: Health assessment questionnaire
GC: Glucocorticoids
